# The Effect of Information Provision on Public Consensus about Climate Change

**DOI:** 10.1371/journal.pone.0151469

**Published:** 2016-04-11

**Authors:** Tatyana Deryugina, Olga Shurchkov

**Affiliations:** 1 Department of Finance, University of Illinois, Urbana-Champaign, IL, United States of America; 2 Department of Economics, Wellesley College, Wellesley, MA, United States of America; University of Waikato (National Institute of Water and Atmospheric Research), NEW ZEALAND

## Abstract

Despite over 20 years of research and scientific consensus on the topic, climate change continues to be a politically polarizing issue. We conducted a survey experiment to test whether providing the public with information on the exact extent of scientific agreement about the occurrence and causes of climate change affects respondents’ own beliefs and bridges the divide between conservatives and liberals. First, we show that the public significantly underestimated the extent of the scientific consensus. We then find that those given concrete information about scientists’ views were more likely to report believing that climate change was already underway and that it was caused by humans. However, their beliefs about the necessity of making policy decisions and their willingness to donate money to combat climate change were not affected. Information provision affected liberals, moderates, and conservatives similarly, implying that the gap in beliefs between liberals and conservatives is not likely to be bridged by information treatments similar to the one we study. Finally, we conducted a 6-month follow-up with respondents to see if the treatment effect persisted; the results were statistically inconclusive.

## Introduction

Climate change generates much public disagreement, despite the broad consensus of scientists that it is a real phenomenon caused by human emissions of greenhouse gases [1–3]. Because climate change is a high-profile issue, implementing meaningful policies to address it will almost surely require a significant degree of public consensus. However, only 50% of US adults believe that climate change is mostly man-made, compared to 87% of scientists [3]. Moreover, differences between the beliefs of liberals and conservatives are stark and have recently widened. In 2001, 60% of Democrats and about 50% of Republicans believed the effects of global warming have already begun to happen; by 2008, that figure was 75% of Democrats compared to 42% of Republicans [4]. Thus, the current gap between the consensus among scientists and the public consensus on this issue is driven largely by conservatives [3]. In the US Congress, one political party can prevent another from passing legislation unless the latter has a supermajority. Because of this, the ideological divide on climate change can hinder public policy even if that policy has the support of the majority of Americans. Thus, whether and how the overall public consensus on this issue can be brought closer to that of scientists, and how the ideological divide can be bridged are both important public policy questions.

There is a strong correlation between individuals’ own views on global warming and their beliefs about scientists’ views on the topic [5], but whether this means that people rely on their perceptions of scientists’ beliefs to form their own is unclear. For example, it is possible that people misstate their beliefs about scientists’ views to justify their own opinion about climate change: an experiment [6] shows that subjects who make selfish choices in social dilemmas are also likely to report inaccurate beliefs to justify their own selfishness. However, the absence of people’s disagreement with perceived scientific views suggests another (not necessarily mutually exclusive) hypothesis: that beliefs can be affected by providing respondents with objective information about the scientific consensus. In this paper, we investigate whether such information can steer the public toward a consensus on the science of global climate change.

We conducted a survey experiment to estimate the causal effects of providing objective information about climate scientists’ views on climate change on respondents’ own beliefs and their willingness to contribute to causes that aim to counter climate change. The treatment group received precise information about the beliefs of US scientists who have published articles in top climate journals [1], while the control group received no information. A third group received vague information about the scientific consensus to test whether simply drawing subjects’ attention to the fact that a consensus exists without providing specific evidence would have a meaningful impact on stated beliefs. We then assessed the treated group’s perception of the credibility of the information and elicited all respondents’ beliefs about various aspects of climate change. Finally, we followed up with all respondents 6 months after the initial treatment without any additional interventions to test whether the treatment effect persisted.

Research has shown that providing objective information alters behavior in contexts other than climate change [7–8]. Although some experimental studies on the factors affecting beliefs about climate change exist [9–13], ours is one of the first to test the effects of providing objective information about the scientific consensus, to assess the public’s perception of the credibility of such information, and to gauge the persistence of the effects over time. An exception is a recent information experiment conducted around the same time as this study [14–15]. Researchers have argued that familiarity with the scientific consensus is a necessary prerequisite for the public to make informed decisions [16–17]. However, whether or not information provision would actually affect beliefs is unclear if there is substantial mistrust toward information about climate change in general [16]. Our survey experiment helps illuminate the importance of both objective information and trust in communications about climate change.

## Survey Experiment

### Survey Methodology

The survey responses were collected through e-mail by Marketing Systems Group (MSG) and SurveySavvy, a professional survey company. The baseline survey was conducted between April 9 and April 17, 2013. A follow-up survey was conducted 6 months later, between September 30 and October 28, 2013. Subjects were recruited from SurveySavvy’s current pool of survey respondents. SurveySavvy recruits subjects into the survey pool through the company’s website and a proprietary system of online referrals. In addition, SurveySavvy reaches out to groups that are under-represented in their pool via e-mail and telephone, both landline and cellular, in order to ensure that the survey pool is close to nationally representative. Recruitment information screen shots, the text of the e-mail sent to the prospective participants, and a recruitment flowchart can be found in S1 Text.

The study received approval from the University of Illinois, Urbana-Champaign’s Institutional Review Board for non-biomedical human subject research. The only criterion for participating in the baseline survey was for respondents to be 18 years of age or older (self-reported) and located in the United States. An additional criterion for the 6-month follow-up survey was for respondents to have completed the baseline survey. No vulnerable populations were targeted, and none of the subjects were likely to be associated with the researcher. All participants read an informed consent form prior to starting the survey. This form informed the participants about the purpose of the study, the duration of the survey (approximately 10 minutes), the incentive for survey completion (the opportunity to win one of six $50 prizes in addition to a financial incentive provided by SurveySavvy), the existence of a 6-months follow-up survey, the fact that participation was completely voluntary and could be terminated at any point, and the anonymity and processing of individual data. The form also provided investigator contact information. Subjects were not exposed to any risks or deception and were able to quit the survey at any point. At the end of the form, the participants could either terminate participation or click “Yes” to continue to the survey. Due to the online nature of the survey and minimal risk to the subjects, IRB granted a waiver of written informed consent. The full informed consent forms for the initial survey and 6-month follow-up are available in S2 Text. The survey did not ask for personally identifiable information, such as name or email. In order to ensure complete anonymity, results are presented in the aggregate across all survey respondents. Finally, the data are stored on the hard drives of the researchers’ password-protected computers.

The “closed” electronic survey was sent to prospective participants via e-mail that provided a unique link to the survey. The survey was programmed online using SurveyMonkey.com. The uniqueness of the link allowed duplicate responses to be identified and dropped. Thus, it was not necessary to use cookies to track respondents. Similar questions were grouped on the same screen. There were a total of 14 screens in the baseline survey: a screen with the informed consent information, 12 question screens, and a screen thanking respondents for their participation. There were a total of 13 screens in the 6-month follow-up survey: a screen with the informed consent information, 11 question screens, and a screen thanking respondents for their participation. Participants could not go back to review or change their answers to previous questions in either survey. The order of questions was kept the same across all participants, and adaptive questioning was not applicable. The order of answers within each multiple choice question was randomized, but the order of the questions themselves did not vary. The full survey text is available in S3 Text. Prior to running the full-scale survey, the usability and technical functionality of the electronic questionnaire was tested with 5 volunteers. A pilot survey with 201 respondents was conducted to test whether questions were well-formulated. Because there was no follow-up with the pilot respondents, they are excluded from the results reported in this paper. However, their inclusion would not significantly change the baseline results.

Out of the 2,484 invited participants, 1,593 respondents initiated and 1,300 completed the baseline survey (for a participation rate of 64% and a completion rate of 82%). Because the survey was sent to prospective participants via e-mail, the “view rate” is equal to the participation rate in our survey; we did not have the capability to track whether respondents opened the email or not. Out of the 1,300 participants who completed the baseline survey, 886 initiated and 747 completed the follow-up survey (for a participation rate of 68% and a completion rate of 84%). While there were some statistically significant differences between the characteristics of the initial sample and of those who completed the follow-up survey (see S4 Text), there was no differential attrition by treatment status.

Only completed questionnaires were analyzed. We did not measure the time respondents spent answering the questions; all completed questionnaires were used in our analysis. No statistical correction methods were applied to either the baseline or the follow-up samples. Summary statistics of respondent characteristics can be found in S4 Text. Our sample was broadly similar to the US as a whole, with the exception of education: a significantly higher share of our respondents had a bachelor’s degree or higher [18].

### Treatments

In the baseline survey, all respondents first answered questions that elicited general attitudes toward climate change science and assessed knowledge about climate science (e.g., being able to name greenhouse gases). Next, the participants were randomly assigned to one of three equal groups. The control group received no information to provide benchmark measurements of (a) beliefs about the scientific consensus and (b) the relationships between beliefs about the scientific consensus, beliefs about climate change, and political ideology.

The treatment group viewed a screen with the following passage that informed them about scientists’ beliefs about climate change (“hard information treatment,” hereafter HI). To present the information as objectively as possible, we used exact quotes from the survey of scientists from which the information was obtained.

*“There is strong scientific consensus about the occurrence and cause of global warming*. *In a 2005 academic survey of US scientists who have published articles in the top climate science journals*, *94 percent of scientists agreed with the statement "Scientists can say with great certainty that global warming is a process that is already underway*.*" 88 percent agreed with the statement "Scientists can say with great certainty that human activities are accelerating global warming*.*" 9 percent agreed with the statement "There is enough scientific uncertainty about the rate and extent of global warming and climate change that there is no need for immediate policy decisions*.*”*

*Source*: *Rosenberg S*., *Vedlitz A*., *Cowman D*., *and S*. *Zahran*. *2010*. *"Climate change*: *a profile of U*.*S*. *climate scientists' perspectives"*, *Climatic Change 101 (3–4)*: *pp*. *663–668*.

The third group viewed a screen with similarly worded but vague information about climate change (“soft information treatment,” hereafter SI).

*“There is strong scientific consensus about the occurrence and cause of global warming*. *The overwhelming majority of scientists agree that global warming is already underway and that human activities are accelerating it*. *Moreover*, *most scientists agree that there is enough certainty about the rate and extent of global warming to warrant immediate policy decisions*.*”*

This treatment allows us to test whether simply drawing respondents’ attention to the fact that a consensus exists, without providing any concrete evidence, affects beliefs. Here, we are agnostic as to whether the impact on beliefs, if any, is due to (a) being reminded about something subjects already knew (the salience channel), (b) the novelty of this vague information to at least some subjects, or (c) both.

Following the information screen, the HI group answered questions about the credibility of the information we provided in order to gauge their level of skepticism. The SI and the control groups answered questions about their perception of what scientists believe. It is possible that by asking HI respondents about the credibility of the information, we caused some subjects to think that the information was incorrect, undermining its credibility. If that were the case, our estimated treatment effect would have been *smaller* than if we had asked the questions about the credibility of the information last.

All groups then answered the same key questions of interest, which elicited respondents’ own beliefs about climate change and their willingness to sacrifice a portion of a monetary prize in order to contribute toward a cause that counters climate change by promoting energy efficiency. The wording of the statements was chosen to correspond closely to the scientist survey in [1]. Specifically, questions regarding beliefs about climate change were phrased in the following way.

*In your opinion*, *what is the probability that each of the following is true*, *out of 100%*?

*1. Global warming is a process that is already underway*.

*2. Human activities are accelerating global warming*.

*3. There is enough scientific uncertainty about the rate and extent of global warming and climate change that there is no need for immediate policy decisions*.

*By how many degrees Fahrenheit do you expect temperatures on earth to rise or fall by the year 2050*, *on average*? *(a change of 1 degree Fahrenheit is about equal to a change of 0*.*56 degrees Celsius)*

*What do you think is the probability that the temperature will increase/decrease by at least 2*.*5/5 degrees Fahrenheit by 2050*? *(4 questions total)*

We then asked the following question to gauge whether beliefs translate into willingness to pay.

*After completing the survey*, *you will be entered in a drawing for one of six $50 prizes*. *You have the option to send part of your winnings to Alliance to Save Energy*, *a nonprofit organization that is working to prevent the onset of climate change through promoting energy efficiency*. *Should you win*, *the amount will be deducted prior to you receiving the prize money and anonymously sent to Alliance to Save Energy*.

*If you win one of the prizes*, *how much of your winnings do you want sent to Alliance to Save Energy*? *Enter a dollar amount between $0 and $50*.

The decision about donations was thus incentivized by asking respondents how much of their own potential winnings they would be willing to sacrifice to address the issue of climate change. We selected a relatively unknown organization, whose methods (promoting energy efficiency) are relatively uncontroversial, conditional on being involved in climate change. By specifying that the donation would be “anonymously sent”, we tried to reduce the possibility that the study would be viewed as being associated with or conducted by that organization. However, because this question was last among those used to elicit climate change beliefs, any differential skepticism on behalf of the treated and control groups at this point could not have affected answers to the previous questions.

The survey concluded with detailed demographic questions including ideology and educational attainment. Finally, in order to see whether the treatment produced lasting effects, we conducted a follow-up survey 6 months later. The key questions were identical to the baseline except that the follow-up survey did not contain any treatment or treatment-specific questions.

## Empirical Strategy

Due to the randomized nature of our study, the basic empirical strategy is straightforward: we compare the beliefs of respondents who were and were not exposed to the treatment. To increase efficiency, we also control for respondent characteristics. Specifically, we estimate the following equation, using ordinary least squares (OLS) regression:

$$Belief_{i}=\alpha +\gamma Treat_{i}+\theta SoftInfo_{i}+X_{i}^{\prime }\beta +\varepsilon_{i}$$ (1)

The variable *Belief*_*i*_ is the reported belief of respondent *i* about some aspect of climate change, such as whether or not it is caused by humans. We use the indicator *Treat*_*i*_ to denote treated individuals and the indicator *SoftInfo*_*i*_ to denote those who were in the soft information group. Finally, because controlling for covariates even in an experimental setting can improve the precision of the estimates [19], our empirical model includes ***X***_***i***_—a vector of respondent characteristics, including age, age squared, gender, as well as sets of race, income, employment, education, and ideology indicators.

Thus, *γ* is the difference in the average outcome between the treated and control groups, conditional on respondent characteristics. Similarly, *θ* measures the difference between the beliefs of the soft information group and the control. Testing whether *γ* = *θ* reveals whether the effect of information is coming from its content or is simply due to the fact that subjects were told that a scientific consensus exists without additional evidence.

To examine treatment heterogeneity, we interact the treatment indicator with respondent characteristics, such as their ideology, education, and the degree to which they trust scientists. The last measure was elicited before the treatment and thus should not be affected by it.

## Results

### Basic findings

The responses of the control group about scientists’ views on climate change reveal that the public underestimates the degree of scientific consensus, as measured by the survey of scientists we use to provide information to the treated group [1]. In particular, respondents in the control group believe that only 72% of scientists agree that global warming is a process that is already underway (different from 95%, the percentage of scientists agreeing with the same statement, at *p* < 0.001), that only 69% of scientists believe that human activities are accelerating global warming (different from 88% at *p* < 0.001), and that 32% of scientists would say that there is no need for immediate policy decisions (different from 9% at *p* < 0.001).

We also find a great degree of skepticism toward the information shown to the HI treatment group. Almost two-thirds (65%) of the treated group did not think the information from the scientist survey was accurately representing the views of all scientists who were knowledgeable about climate change. Only about 20% of the skeptical respondents thought that participating scientists misstated their true views. The skepticism largely stems from the concern that the scientists polled (“US scientists who published in top journals”) were not representative of all scientists knowledgeable about climate change: 85% of skeptical respondents chose that as one of the reasons for thinking that the information was inaccurate. Being unemployed, liberal or conservative (versus moderate) were the only significant predictors of not trusting the information from the survey of US climate scientists.

Those in the treatment group who were skeptical of the information also believed that scientists were less certain about climate change than the control group (by about 4 percentage points, on average). On the other hand, the control group did not think that there would be significant differences in the answers by the following two groups of scientists: (a) all scientists knowledgeable about climate change, and (b) only US scientists who published in top journals. The fact that the treated respondents reported that (b) would not be representative of (a) while the control group does not expect there to be significant differences between them suggests that some people in our sample may suffer from “self-justification” bias. “Self-justification” refers to the phenomenon of justifying one’s behavior or beliefs when facing evidence that is inconsistent with them [20]. For example, in our case, the self-justification bias may explain why some respondents continue to believe that climate change is not caused by humans when facing the fact that most scientists believe that it is. Self-justification bias may also be responsible for the disconnect described above: one way to justify continuing to believe that climate change is not occurring or is not caused by humans in light of the scientific consensus information is to claim that the survey does not accurately reflect all scientists’ views. Alternatively, it may be that learning about US climate scientists’ views on climate change caused some of the treated respondents to rationally conclude that these scientists’ views differ from the views of all scientists knowledgeable about climate change. A more detailed analysis of skepticism can be found in S5 Text.

### The immediate effect of information

In this section, we econometrically estimate the short-run effects of information on respondents’ beliefs about climate change and their willingness to contribute toward preventing the onset of climate change. Table 1 shows the treatment effect of information on our various measures of beliefs, conditional on extensive respondent controls, including age, age squared, and indicators for gender, race, employment status, education, income, and political ideology. All specifications also include an indicator for soft information treatment. All subsequent specifications include these controls, unless otherwise specified. Excluding the soft information indicator or doing the analysis using the treated and control groups only does not substantively change the results. All specifications cluster standard errors at the state level. The unconditional mean of each dependent variable is shown in the row “Dep. var. mean”.

**Table 1:** Average short-run effects of information on beliefs about climate change.

	Underway	Caused by Humans	Prob Chg > 2.5	Prob Chg > 5	Prob Chg < -5	Prob Chg < -2.5	Change by 2050
Hard info	5.91***	5.09**	5.80**	5.92***	1.63	2.02	-0.02
	(1.67)	(1.91)	(2.67)	(2.10)	(1.19)	(1.47)	(0.41)
Soft info	0.30	0.94	2.15	2.92	0.51	0.84	0.11
	(1.39)	(1.67)	(2.05)	(2.13)	(1.37)	(1.42)	(0.45)
Soft = hard p-val	<0.001	0.05	0.16	0.21	0.42	0.47	0.76
Dep. var. mean	74.29	68.04	56.43	41.33	14.04	18.74	5.05
Observations	1,259	1,259	1,259	1,259	1,259	1,259	1,258
R-squared	0.12	0.16	0.10	0.12	0.05	0.04	0.08

Standard errors clustered at the state level in parentheses; all specifications include controls for age, age squared, gender, race, employment status, education, income, and political ideology. Significance levels:

*10%

**5%

***1%

Columns (1) and (2) reveal a significant treatment effect: receiving the information about scientists’ beliefs raises respondents’ beliefs that climate change is already underway and that it has been caused by human activity by 6 and 5 percentage points, respectively. The beliefs of those in the soft information group, on the other hand, do not differ significantly from those in the control group, which suggests that beliefs are impacted by the content and evidence, rather than just by the availability of vague information.

Columns (3)-(6) report the effects of information on expected temperature changes due to climate change by year 2050. Overall, the treatment group believes that an increase in temperatures of 2.5 or 5 degrees Fahrenheit is about 6 percentage points more likely than the control group (Columns 3 and 4). On the other hand, there is no difference between the treated and control group in terms of the probability that temperatures *decrease* over this time (Columns 5 and 6). Finally, the treatment group’s answers about the expected change in temperature by 2050 in degrees Fahrenheit does not differ significantly from the control group (Column 7).

Table 2 investigates whether the effect of information on beliefs translates into policy preferences or actions. Column (1) estimates the effect on information on the belief that “there is enough scientific uncertainty about the rate and extent of global warming and climate change that there is no need for immediate policy decisions”. We find no significant treatment effect. This is consistent with a model where people look to climate scientists for objective scientific information but not public policy recommendations, which also require economic (i.e. cost-benefit) and ethical considerations. The point estimates of the effect of information on the willingness to donate some of the potential winnings (Column 2) and on the donation amount (Column 3) are positive, but not significant. We find a significant difference between the hard information treatment and the soft information treatment, however. In fact, vague information seems to decrease donations. This finding is consistent with previous work by [21] who find that information reduces misperceptions that vaccines cause autism but nonetheless decreases intent to vaccinate among parents who had the least favorable vaccine attitudes.

**Table 2:** Average short-run effects of information on policy actions.

	Beliefs about Policy	Prob. Donate	Donation Amount
Hard info	-0.83	0.09	0.78
	(2.00)	(0.08)	(0.77)
Soft info	-0.61	-0.09	-1.05*
	(1.98)	(0.07)	(0.53)
Soft = hard p-val	0.90	0.03	0.02
Dep. var. mean	33.58	0.50	8.72
Observations	1,259	1,259	1,259
R-squared	0.09	0.12	0.05

Standard errors clustered at the state level in parentheses; all specifications include controls for age, age squared, gender, race, employment status, education, income, and political ideology. Significance levels:

*10%

**5%

***1%

### Heterogeneity in the short-run effects

Next, we decompose the treatment effect according to respondents’ political ideology, education, and climate change knowledge. Table 3 shows the effects of information broken down by reported political ideology. In Column 1, the belief that climate change is already underway seems to be most strongly affected by information among the liberals and the conservatives. However, the standard errors are large and we are unable to reject that the three coefficients are significantly different from one another. Columns 2, 4, and 5 suggest that information most strongly affects moderates’ beliefs about humans causing climate change and about the probability of temperature increases, although once again, we are unable to reject that the three ideological groups respond to information in an identical manner. Beliefs about policy actions are not significantly affected by information for any of the ideological groups (Column 3). Finally, treated conservatives donate marginally more to climate change causes, although we fail to reject that the point estimates for the three groups are equal (Column 6).

**Table 3:** Short-run effects of information by ideology.

	Underway	Humans	Policy	Pr(Chg > 2.5)	Pr(Chg > 5)	Don. Am't
H.I.: liberal	6.45***	2.27	2.14	0.81	0.33	-0.79
	(2.24)	(2.61)	(4.28)	(3.55)	(3.38)	(2.15)
H.I.: moderate	4.21	8.67***	-3.72	10.77**	11.14***	0.71
	(2.65)	(3.16)	(2.82)	(4.59)	(3.83)	(2.00)
H.I.: conservative	7.79**	2.52	0.81	2.90	2.90	1.86*
	(3.42)	(3.74)	(3.59)	(3.41)	(3.62)	(0.95)
S.I.: liberal	1.27	1.56	3.51	-2.57	-0.15	-1.78
	(2.68)	(2.21)	(4.36)	(3.90)	(6.48)	(1.87)
S.I.: moderate	0.15	3.81	-6.20*	6.21**	7.60***	-1.34
	(2.44)	(3.06)	(3.69)	(2.72)	(2.81)	(1.45)
S.I.: conservative	-0.30	-3.66	4.72	0.13	-1.45	-0.13
	(4.02)	(3.29)	(4.05)	(3.92)	(3.27)	(1.33)
Dep. var. mean	74.29	68.04	33.58	56.43	41.33	8.72
Observations	1,259	1,259	1,259	1,259	1,259	1,259
R-squared	0.12	0.16	0.09	0.11	0.13	0.05

Standard errors clustered at the state level in parentheses; all specifications include controls for age, age squared, gender, race, employment status, education, and income. Significance levels:

*10%

**5%

***1%

Table 4 decomposes the effect of information by educational attainment. In our survey, educational attainment is broken up into 8 categories, ranging from less than high school to doctoral degrees (PhD) and professional degrees (MD, JD, DDS, etc.). For the purpose of this analysis, however, we combine the categories into two bins: some college or 2-year degree and below (low education) and 4-year college degree and above (high education). We find that receiving hard information significantly impacts the highly educated in terms of increasing their beliefs about climate change being already underway, beliefs that there would be an increase in temperature by more than 5 degrees Fahrenheit by 2050, and the amount donated to climate change causes (Columns 1, 5, and 6). On the other, the low education treatment group is more likely to believe that climate change has been caused by human activities (Column 2). However, once again, in all cases, we cannot reject that the high and the low education groups respond to the treatment in the same way.

**Table 4:** Short-run effects of information by education.

	Underway	Humans	Policy	Pr (Chg > 2.5)	Pr (Chg > 5)	Don. Am't
H.I.: low educ	2.12	6.38**	-2.06	3.68	3.68	-0.32
	(3.17)	(2.74)	(3.45)	(3.34)	(3.37)	(1.35)
H.I.: high educ	10.47***	3.79	1.62	6.82	7.79***	2.54**
	(3.43)	(3.38)	(3.34)	(4.73)	(2.84)	(1.18)
S.I.: low education	0.93	2.56	-2.51	2.27	2.72	-1.50
	(2.66)	(2.50)	(2.95)	(3.30)	(2.64)	(0.90)
S.I.: high education	1.73	1.01	0.54	3.67	3.90	-0.40
	(3.54)	(3.57)	(2.90)	(3.52)	(3.77)	(1.16)
Dep. var. mean	74.27	68.08	33.63	56.31	41.47	8.64
Observations	1,223	1,223	1,223	1,223	1,223	1,223
R-squared	0.11	0.15	0.08	0.09	0.11	0.04

Standard errors clustered at the state level in parentheses; all specifications include controls for age, age squared, gender, race, employment status, income, and political ideology. Significance levels:

*10%

**5%

***1%

Finally, Table 5 breaks down the effect of information by prior knowledge about climate science. We measure this knowledge by the number of correct answers to questions about climate science, such as being able to name greenhouse gases (see detailed survey in S4 Text). The high knowledge group answered more than two questions correctly, while the low knowledge group answered 2 or fewer questions. Overall, the precision of our estimates does not allow us to statistically distinguish between the two groups. We find that both high and low knowledge respondents are positively affect by information in terms of their beliefs about climate change being underway (Column 1) and the probability that global temperature will rise by more than 5 degree Fahrenheit by 2050 (Column 5). The beliefs that climate change is caused by humans and that temperature would rise by more than 2.5 degree Fahrenheit by 2050 are significantly increased for the treated low knowledge respondents (Columns 2 and 4), although the coefficients for the high knowledge group are not significantly different.

**Table 5:** Short-run effects of information by knowledge.

	Underway	Humans	Policy	Pr (Chg > 2.5)	Pr (Chg > 5)	Don. Am't
H.I.: low know	6.04*	5.69**	-0.87	8.11***	5.75**	0.32
	(3.10)	(2.62)	(3.46)	(2.82)	(2.79)	(1.30)
H.I.: high know	5.45**	4.33	-0.58	3.52	5.85**	1.15
	(2.28)	(2.60)	(2.36)	(3.90)	(2.85)	(1.74)
S.I.: low know	-0.83	-3.25	-1.50	0.98	0.33	-1.11
	(1.87)	(2.18)	(3.30)	(2.55)	(2.71)	(0.98)
S.I.: high know	1.80	5.31*	0.03	3.69	5.67	-0.98
	(2.31)	(2.65)	(2.77)	(2.74)	(3.51)	(1.10)
Dep. var. mean	74.29	68.04	33.58	56.43	41.33	8.72
Observations	1,259	1,259	1,259	1,259	1,259	1,259
R-squared	0.16	0.19	0.09	0.13	0.13	0.05

Standard errors clustered at the state level in parentheses; all specifications include controls for age, age squared, gender, race, employment status, income, education and political ideology. Significance levels:

*10%

**5%

***1%

### Long-run effects of information

A 6-month follow-up survey allows us to gauge whether the short-run effects persist over time. In the follow-up, we ask the same questions about beliefs about climate change as we do in the baseline survey, but do not re-administer the treatment. Tables 6 and 7 summarize the results. Although the point estimates on the hard information treatment indicator are positive, we no longer find a significant effect of information, except for the probability that temperatures increase or decrease by 2.5 degrees Fahrenheit or more (Table 6, Columns 3 and 6). At the same time, the standard errors are large, so that we cannot reject the hypothesis that the treatment effects are persistent. Because only roughly half of the original respondents completed the follow-up survey, our small sample size may mask the existence of a long-run effect (or lack thereof).

**Table 6:** Average long-run effects of information on beliefs about climate change.

	Underway	Caused by Humans	Prob Chg > 2.5	Prob Chg > 5	Prob Chg < -5	Prob Chg < -2.5	Change by 2050
Hard info	3.27	2.49	6.19**	4.25	3.50	4.89**	0.15
	(2.58)	(2.48)	(2.51)	(2.70)	(2.33)	(2.22)	(0.51)
Soft info	-0.70	-0.60	3.96	1.76	-0.96	-0.18	-0.14
	(2.76)	(2.74)	(3.07)	(2.47)	(1.90)	(2.18)	(0.43)
Soft = hard p-val	0.17	0.33	0.55	0.43	0.04	0.06	0.51
Dep. var. mean	72.59	68.33	57.24	40.89	13.12	18.93	4.53
Observations	746	746	728	728	728	727	728
R-squared	0.12	0.13	0.10	0.13	0.09	0.06	0.06

Standard errors clustered at the state level in parentheses; all specifications include controls for age, age squared, gender, race, employment status, income, education and political ideology. Significance levels:

*10%

**5%

***1%

**Table 7:** Average long-run effects of information on policy actions.

	Beliefs about Policy	Prob. Donate	Donation Am't
Hard info	-1.13	-0.14	-0.77
	(2.26)	(0.12)	(1.20)
Soft info	-1.57	-0.04	-0.79
	(2.25)	(0.10)	(1.16)
Soft = hard p-val	0.86	0.39	0.99
Dep. var. mean	33.25	0.44	8.08
Observations	746	726	726
R-squared	0.12	0.06	0.06

Standard errors clustered at the state level in parentheses; all specifications include controls for age, age squared, gender, race, employment status, income, education and political ideology. Significance levels:

*10%

**5%

***1%

Power analysis reveals that, holding constant the estimated effect on the beliefs about whether climate change is occurring (Column 1 of Table 6), we would need about 2,600 observations in total (1,300 treated and 1,300 control) to have an 80% chance of rejecting a null hypothesis of no treatment effect with 95% confidence. For the beliefs about whether humans are causing climate change (Column 2 of Table 6), we would need about 5,200 individuals to do so. If these two long-run estimates were significant, they would show that about 50–55% of the original effect of the “hard information” treatment persists for at least six months. Given the magnitude of these estimates, the simplicity of the treatment (i.e., each treated respondent read only one paragraph), and the relative lack of research about whether or not information treatment effects persist, obtaining a larger sample size to see whether the long-run effects are indeed significant should be a fruitful area for future research.

## Discussion

The results of our survey experiment indicate that objective information about the scientific consensus has a short-run effect on the public’s beliefs about climate change. However, we do not observe an increase in either the public’s view that policy action is warranted or their willingness to donate real funds toward climate change causes. The lack of updating based on objective information in this context is consistent with a number of explanations, including strong priors, self-justification bias, selective attention, cultural norms, partisan bias, and information discounting [16,22–24].

The experiment also reveals a great degree of skepticism among the treated respondents toward the information about what scientists believe. The skeptics among the treated also believed that scientists were less certain about climate change relative to the control group. It is also possible that this “information discounting” stems from the partisan bias associated with the issue of climate change [24]. That is, conservative respondents may be more dismissive of the information because the Republican Party is typically skeptical of climate change. Finally, our findings are also consistent with the randomized survey evidence of skepticism toward information in other contexts, such as views and policy preferences for taxation and redistribution [25]. To shed light on the optimal design of information provision, further exploration of the different mechanisms behind the observed updating patterns would be useful.

Numerous correlational studies have explored the relationships between climate change beliefs and various individual characteristics, such as political ideology, age, and education [17,26]. However, correlational findings often have ambiguous causal implications. For example, researchers have found that individuals who underestimate the degree of scientific consensus are also less likely to support policies that would combat climate change [5,27]. This finding has at least two possible explanations. One is that becoming informed about the scientific consensus increases support for policies aimed at combating climate change. Another is that those who are most concerned about climate change seek out more knowledge about the scientific consensus, thus becoming more informed. Our findings suggest that the former explanation is unlikely, at least in our sample: we find no evidence that providing information about the scientific consensus affects policy preferences or raises willingness to pay to combat climate change.

Our sample was not large enough to make further conclusions about the heterogeneity of the effects by demographic characteristics of the respondents, such as ideology, education, or prior climate change knowledge. We are also unable to make definitive conclusions about the long-run persistence of informational effects. Replicating the experiment with a larger sample size would be a fruitful path for future research.

## Supporting Information

S1 Text Detailed information on how survey participants are recruited by SurveySavvy. (PDF)

S2 Text Exact text of the online consent forms for the initial and follow-up surveys. (PDF)

S3 Text Exact text of the initial and follow-up surveys. (PDF)

S4 Text Demographic and economic characteristics of survey respondents. (PDF)

S5 Text Details on analysis of skepticism toward the scientific consensus information. (PDF)
