# Supplementary material for: The Effect of Information Provision on Public Consensus about Climate Change
Source: PLoS One. 2016 Apr 11;11(4):e0151469. doi: 10.1371/journal.pone.0151469 (PMC4827814; doi:10.1371/journal.pone.0151469)
Supplement: S1 Text — (PDF) [file pone.0151469.s001.pdf]

S1. Recruitment Procedures

SurveySavvy recruits subjects into the survey pool through the company's website and a system of online referrals from others. In addition, SurveySavvy reaches out to groups that are under-represented in their pool via phone and e-mail, in order to ensure that the survey pool is close to nationally representative. See Figures A-C for screen shots taken from the company's webpage. See Figure D for a flowchart that depicts recruitment and randomization into our study.

Panelists receive a personally encrypted link to each study in their email invitation which takes them to our redirection system. When a member clicks on this link, he or she is directed to the beginning of the survey from the SurveySavvy landing page, regardless of whether the study is hosted on a client's system or on Luth Research's servers. The redirection system also captures the respondent's status as they exit the survey with their disposition code.

A typical invitation reads as follows:

Dear XX XXX,

SurveySavvy invites you to share your opinions in an important new survey.

If you qualify and complete this survey, your SurveySavvy account will be credited $1 within two to six weeks. As always, your responses will be kept confidential, and they will be used for research purposes only.

Estimated length of survey: __ minutes
Incentive: $ __

Use the link below to get started:
http://rd.surveysavvy.com/c.php?i=579042507&k=2865474c1e4f9aaf1c456cd434773168

If you have any problems with this survey, please visit the survey support page at:
https://www.surveysavvy.com/support/index.php?p=21&pid=148231

Have you logged into SurveySavvy.com recently? Come check out our new website at http://www.surveysavvy.com!

Like and Follow us for Exclusive Surveys and Contests!

http://www.facebook.com/surveysavvy
http://www.twitter.com/surveysavvy

Thank you for making SurveySavvy.com the trusted connection between people and research.

Best Regards,
The SurveySavvy.com Team

============================

SurveySavvy.com Anti-Spam Policy

SurveySavvy.com strictly adheres to an anti-spam policy. This message has been sent to you because you are listed as a member in our SurveySavvy.com database.

The intended recipient is sdm519@gmail.com

If you would like to unsubscribe from SurveySavvy.com altogether, no longer receiving surveys, go to: https://www.surveysavvy.com/ss/unsubscribe/u.php?eid=sdm519@gmail.com&jn=L5796

Support Contact Info:
SurveySavvy / Luth Research, LLC.
1365 Fourth Ave.
San Diego, CA 92101 USA
Phone: 888-588-4258

English

SURVEY SAVVY
intelligence adds up

HOME JOIN HOW IT WORKS HELP LOGIN

HOW IT WORKS

It's free, safe and easy!

Online Surveys

Companies are looking for consumer opinions from people like you! Since 1999, SurveySavvy® has been directly connecting you with those companies and pays cash for sharing your opinions. After you join and complete your member profile, we use the information to target various demographic groups according to criteria established by our clients. When your profile matches the basic criteria for a specific survey, we will invite you via email to participate. If you pass the in-depth screening portion of the survey and complete the body of the survey, we will credit your account the amount mentioned in the invitation.

There is no guarantee as to how many surveys you may receive or participate. That is based on your personal profile, what our clients are looking for, and if you fit into any open quota group. There is no real average as to how many invites most people receive. The amount of invitations varies, but you may receive more based on your profile.

Behavioral Research

Launched in 2009, SavvyConnect™ is an easy to use desktop application that unobtrusively includes you in behavioral market research while you browse the Internet. When you install the software client, you help our clients better understand online behavior and can receive supplementary paid surveys for remaining active. Incentives for participating in SavvyConnect™ continue to evolve as we develop the program.

As an added benefit, SavvyConnect™ provides easy and instant access to all of your online survey invitations right on your desktop. Installation of the software is not required to participate in online surveys. The software recognizes the "Private Browsing" and "Incognito Mode" of the major Internet browsers and stops transmitting when these modes are activated.

Member Profile and Portraits

It is important to keep your member profile and portraits current so we can appropriately match you to survey opportunities. Your member profile starts with your registration information, but it is enriched by completing optional portrait surveys. Portrait surveys are a series of additional questions pertaining to a specific subject or aspect of your life that will help us build a more accurate representation of you. Updating your portrait will help you qualify for more research surveys.

Referrals

With one of the most innovative referral programs around, SurveySavvy® continues to grow rapidly through its referral network. The best way to boost your incentives is by simply referring new members. We provide a public referral link you can post on your blog, Facebook, Twitter, or other social media. You can also send private invitations to your friends and family via email.

How does our patented referral payment system work? (U.S. Patent Nos. 6446044, 7194448)

- You get incentives when you complete surveys
- You get incentives when your direct referrals complete surveys.
- You get incentives when your indirect referrals (referrals your direct referrals made) complete surveys.

Sweepstakes and Contests

From time to time we also run contests and special promotions, such as the SurveySavvy Superstar contest where we asked our members to create a video in response to our employee's Thumbs Up, Thumbs Down video or the SurveySavvy Jingle contest.

View the $500 winning SurveySavvy SuperStar contest entry.
View the $500 winning SurveySavvy Jingle contest entry.

Getting Paid

Once you have accumulated more than $1 USD in incentives, you can request payment by using the "Request Payment" tool under the Account section once logged in. Payment is made by check in USD and will be mailed via U.S. post to the residence on file.

Privacy

We work hard to earn and keep your trust by following a strict Privacy Policy. We never sell or share your personally identifiable information; our clients only get to see anonymous or aggregated data. We also enforce a rigorous Anti-SPAM Policy. We'll never send SPAM or any other unsolicited messages to your email address.

Figure A: SurveySavvy "How it works" screen

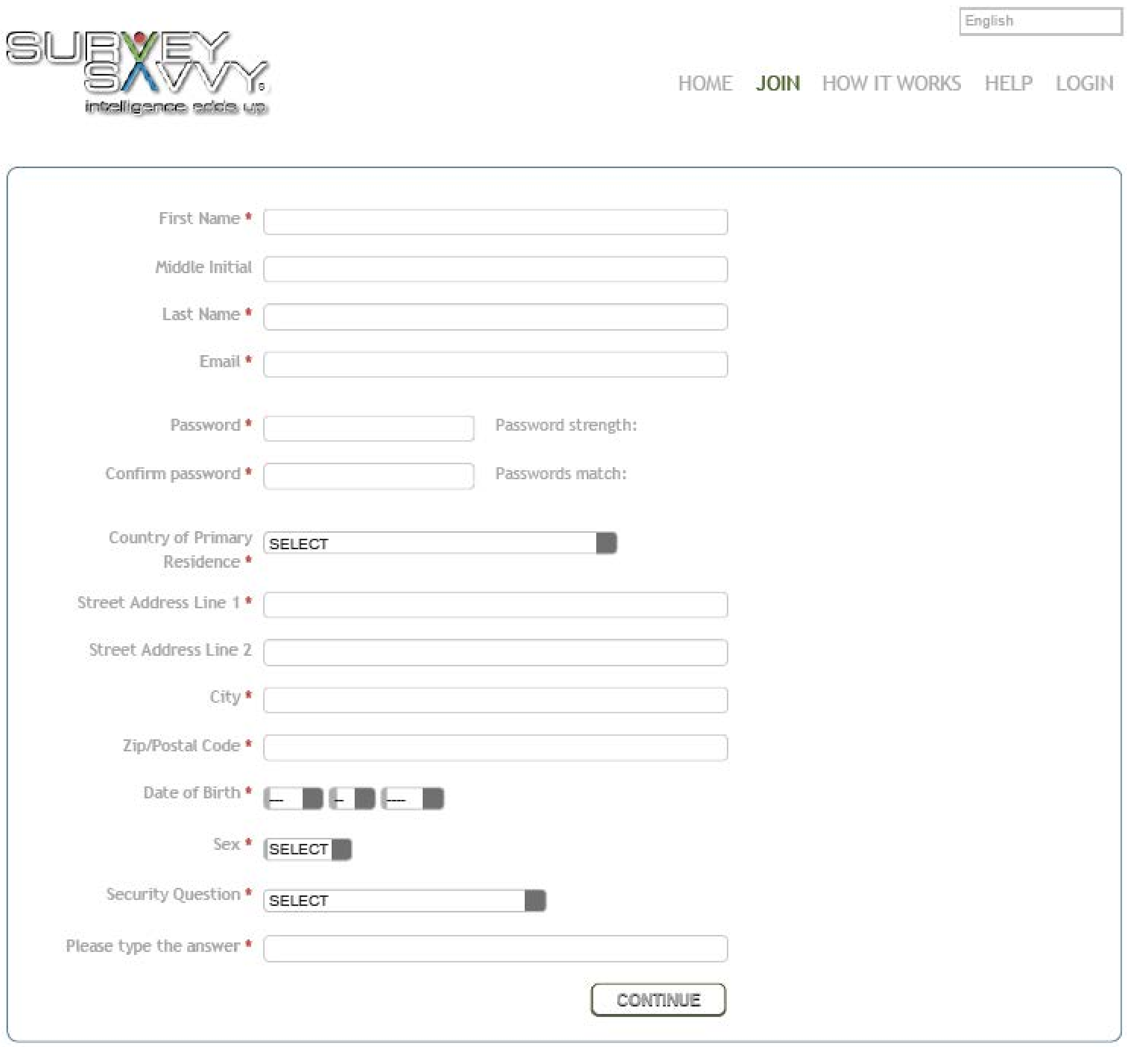

Figure B: SurveySavvy sign-up screen

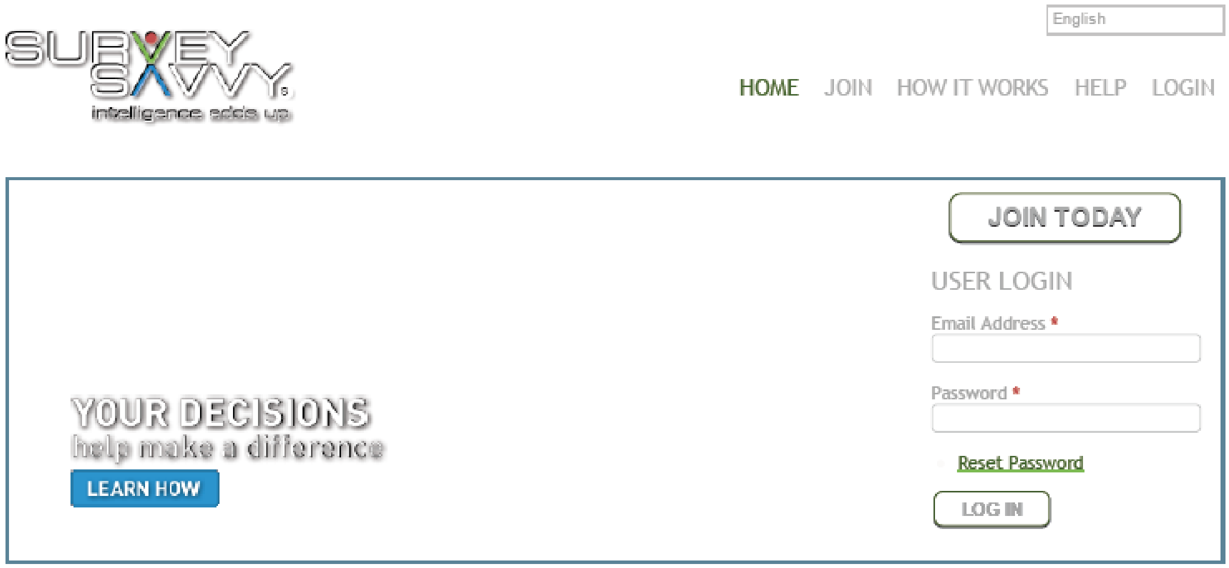

Figure C: SurveySavvy welcome screen and login

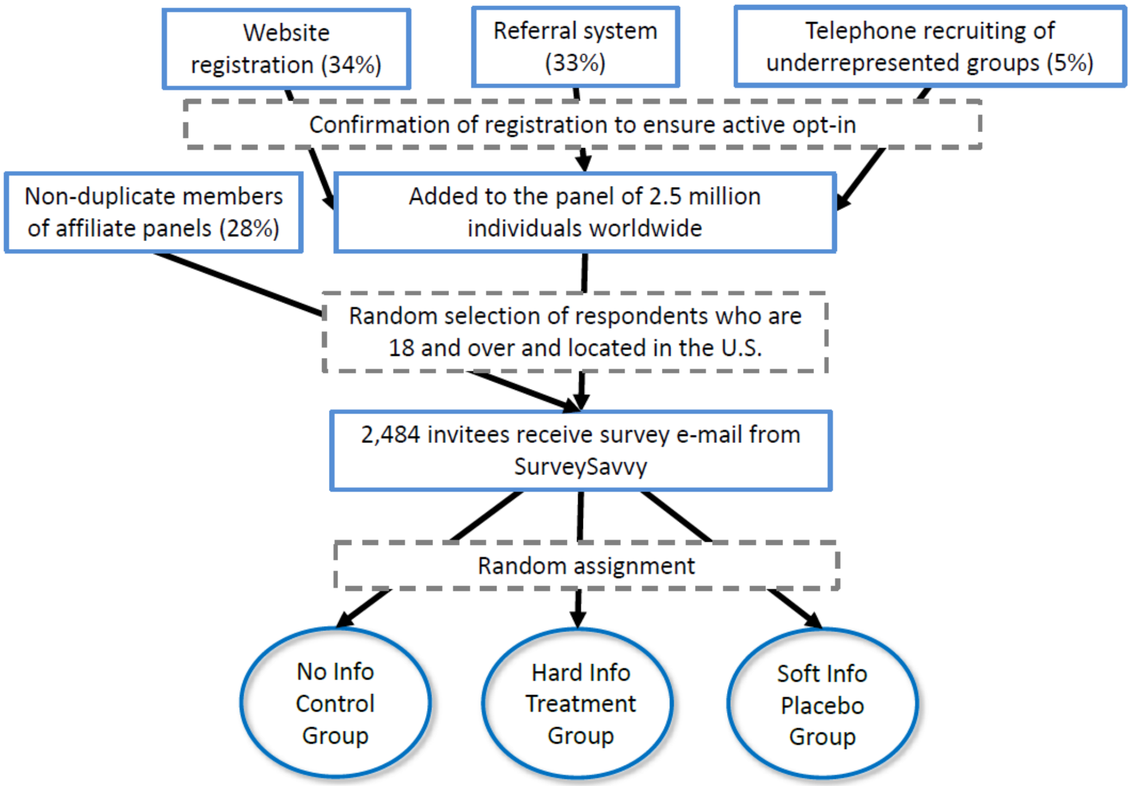

Figure D: Recruitment and Randomization Flowchart
