# Supplementary material for: The Effect of Information Provision on Public Consensus about Climate Change
Source: PLoS One. 2016 Apr 11;11(4):e0151469. doi: 10.1371/journal.pone.0151469 (PMC4827814; doi:10.1371/journal.pone.0151469)
Supplement: S2 Text — (PDF) [file pone.0151469.s002.pdf]

S2. Consent forms

Note: Professor Shurchkov was not involved in the project at the time the data were collected; thus, her name is not mentioned in the consent form.

Initial survey

***Note: The text below will be shown to participants at the beginning of the survey. The signatures on the consent forms, therefore, will be in electronic format.**

Views About Global Warming Survey

You are asked to participate in a research survey conducted by Tatyana Deryugina, Ph.D. and an assistant professor at the University of Illinois, Urbana-Champaign. You were selected as a possible participant in this study because you are at least 18 years old. The purpose of the next few paragraphs is to inform you of the survey and to act as a consent form for purposes of the survey.

The purpose of this study is to understand individuals' views about global warming. There are no direct benefits for the participants beyond getting paid. There are no foreseeable risks or discomforts from participating in this survey.

If you agree to participate in this study, you will be asked to fill out a short survey. Filling out the survey will take about 10 minutes. Following your completion of this survey, you will be entered in a drawing for one of six $50 prizes. Six months from now, you will be contacted again and asked to fill out another short survey. Filling out the second survey will take about 5 minutes. Following your completion of the second survey, you will again be entered in a drawing for one of six $50 prizes.

Your participation in this survey is completely voluntary and you have the right to terminate your participation at any time without penalty. Your participation in this research will be completely anonymous to the researcher. Any results that are reported will only be presented in the aggregate across all respondents to the survey.

If you have any questions or concerns about the research before or after giving consent, please feel free to contact Tatyana Deryugina, University of Illinois at Urbana-Champaign, deryugin@illinois.edu.

If you have any questions about your rights as a participant in this study or any concerns or complaints, please contact the University of Illinois Institutional Review Board at 217-333-2670 (collect calls will be accepted if you identify yourself as a research participant) or via email at irb@illinois.edu.

I have read and understand the above consent form, I certify that I am 18 years old or older, and, by selecting "Yes" below, I indicate my willingness to voluntarily take part in the study.

Six-month follow-up survey

***Note: The text below will be shown to participants at the beginning of the survey. The signatures on the consent forms, therefore, will be in electronic format.**

Views About Global Warming Survey

You are again asked to participate in a research survey conducted by Tatyana Deryugina, Ph.D. of the University of Illinois, Urbana-Champaign. You were selected as a possible participant in this study because you are at least 18 years old. The purpose of the next few paragraphs is to inform you of the survey and to act as a consent form for purposes of the survey.

The purpose of this study is to understand individuals' views about global warming. There are no direct benefits for the participants beyond getting paid. There are no foreseeable risks or discomforts from participating in this survey.

If you agree to continue participating in this study, you will be asked to fill out a short survey. Filling out the survey will take about 5 minutes. Following your completion of this survey, you will be entered in a drawing for one of six $50 prizes.

Your participation in this survey is completely voluntary and you have the right to terminate your participation at any time without penalty. Your participation in this research will be completely anonymous to the researcher. Any results that are reported will only be presented in the aggregate across all respondents to the survey.

If you have any questions or concerns about the research before or after giving consent, please feel free to contact Tatyana Deryugina, University of Illinois at Urbana-Champaign, deryugin@illinois.edu.

If you have any questions about your rights as a participant in this study or any concerns or complaints, please contact the University of Illinois Institutional Review Board at 217-333-2670 (collect calls will be accepted if you identify yourself as a research participant) or via email at irb@illinois.edu.

I have read and understand the above consent form, I certify that I am 18 years old or older, and, by selecting "Yes" below, I indicate my willingness to voluntarily take part in the study.
