# Supplementary material for: The Effect of Information Provision on Public Consensus about Climate Change
Source: PLoS One. 2016 Apr 11;11(4):e0151469. doi: 10.1371/journal.pone.0151469 (PMC4827814; doi:10.1371/journal.pone.0151469)
Supplement: S3 Text — (PDF) [file pone.0151469.s003.pdf]

S3. Survey Protocol

The survey was programmed in SurveyMonkey. Subjects were recruited and paid by Marketing Systems Group (MSG).

Initial survey

Introduction – all subjects

Q1. How much do you agree with each of the following statements? Strongly agree/agree/disagree/strongly disagree. [question order was randomized]

a. I am very interested in what's going on with climate change policy.
b. As a society, we have a moral obligation to leave future generations at least as well off as we were.
c. It's too late to prevent the harmful effects of global warming.
d. Climate scientists subject their findings to a sufficient amount of scrutiny.
e. Climate scientists gain financially from research that supports, rather than contradicts, the theory of global warming.
f. Climate scientists have ideological beliefs that prevent them from carrying out objective research.
g. Climate models are not nearly as reliable as climate scientists claim.

Q2. Which of the following gases is thought to contribute to global warming (select as many as apply)? [answer order was randomized]

a. Methane
b. Carbon dioxide
c. Argon
d. Nitrogen
e. Oxygen
f. Nitrous oxide
g. Ozone

Q3. Which of the following best demonstrates the albedo effect? [answer order was randomized]

a. Ice and other light surfaces reflecting sunlight back into the atmosphere.
b. Prevailing winds causing storm systems to rotate clockwise in the northern hemisphere.
c. Abnormally warm ocean temperatures causing more precipitation in some parts of the world.
d. Atmospheric gases trapping reflected sunlight and re-radiating some of it back toward the planet's surface.

Q4. Which of the following is closest to the current carbon dioxide concentration in the atmosphere? [answer order was randomized]

a. 390-395 parts per million
b. 270-275 parts per million
c. 545-550 parts per million
d. 195-200 parts per million

Soft Information Treatment Addition

There is strong scientific consensus about the occurrence and cause of global warming. The overwhelming majority of scientists agree that global warming is already underway and that human activities are accelerating it. Moreover, most scientists agree that there is enough certainty about the rate and extent of global warming to warrant immediate policy decisions.

Hard Information Treatment Addition

There is strong scientific consensus about the occurrence and cause of global warming. In a 2005 academic survey of US scientists who have published articles in the top climate science journals, 94 percent of scientists agreed with the statement "Scientists can say with great certainty that global warming is a process that is already underway." 88 percent agreed with the statement "Scientists can say with great certainty that human activities are accelerating global warming." 9 percent agreed with the statement "There is enough scientific uncertainty about the rate and extent of global warming and climate change that there is no need for immediate policy decisions."

Source: Rosenberg S., Vedlitz A., Cowman D., and S. Zahran. 2010. "Climate change: a profile of U.S. climate scientists' perspectives", Climatic Change 101 (3–4): pp. 663-668

Hard Information Treatment Only

Q5a. What do you think is the probability that the views of the climate scientists who took the survey above closely reflect the views of <u>all scientists who are knowledgeable about global warming and climate change</u>? (Out of 100%)

Subjects entered number between 0 and 100.

Q5b. If you believe the survey does not reflect the view of <u>all scientists who are knowledgeable about global warming and climate change</u>, why is that (select as many as apply)?

i. Not applicable, believe survey is accurate.
ii. Scientists participating in the survey misstated their true views.
iii. The sample of scientists who participated in the survey does not adequately represent all scientists who are knowledgeable about global warming and climate change.

Q6. Suppose <u>all scientists who are knowledgeable about global warming and climate change</u> were surveyed. What percentage of them do you think would agree with each of the following statements? (Number of scientists out of 100)

i. "Scientists can say with great certainty that global warming is a process that is already underway."
ii. "Scientists can say with great certainty that human activities are accelerating global warming."
iii. "There is enough scientific uncertainty about the rate and extent of global warming and climate change that there is no need for immediate policy decisions."

Control and Soft Information Treatment Only

Q5. Suppose <u>all US scientists who have published articles in top climate journals</u> were surveyed. What percentage of them do you think would agree with each of the following statements? (Number of scientists out of 100)

i. "Scientists can say with great certainty that global warming is a process that is already underway."
ii. "Scientists can say with great certainty that human activities are accelerating global warming."
iii. "There is enough scientific uncertainty about the rate and extent of global warming and climate change that there is no need for immediate policy decisions."

Q6. Now suppose <u>all scientists who are knowledgeable about global warming and climate change</u> were surveyed. What percentage of them do you think would agree with each of the following statements? (Number of scientists out of 100)

i. "Scientists can say with great certainty that global warming is a process that is already underway."
ii. "Scientists can say with great certainty that human activities are accelerating global warming."
iii. "There is enough scientific uncertainty about the rate and extent of global warming and climate change that there is no need for immediate policy decisions."

All Respondents

Q7. In your opinion, what is the probability that each of the following is true, out of 100%?

i. Global warming is a process that is already underway.
ii. Human activities are accelerating global warming.
iii. There is enough scientific uncertainty about the rate and extent of global warming and climate change that there is no need for immediate policy decisions.

For each question, subjects entered a number between 0 and 100.

Q8. By how many degrees Fahrenheit do you expect temperatures on earth to rise or fall by the year 2050, on average? (a change of 1 degree Fahrenheit is about equal to a change of 0.56 degrees Celsius)

Subjects entered a number between -15 and 15

Q9. What do you think is the probability that the temperature will *increase by at least* 2.5 degrees Fahrenheit by 2050?

Subjects entered number between 0 and 100.

Q10. What do you think is the probability that the temperature will *increase by at least* 5 degrees Fahrenheit by 2050?

Subjects entered number between 0 and 100.

Q10. What do you think is the probability that the temperature will *decrease by at least* 2.5 degrees Fahrenheit by 2050?

Subjects entered number between 0 and 100.

Q11. What do you think is the probability that the temperature will *decrease by at least* 5 degrees Fahrenheit by 2050?

Subjects entered number between 0 and 100.

Q12. After completing the survey, you will be entered in a drawing for one of six $50 prizes. You have the option to send part of your winnings to Alliance to Save Energy, a nonprofit organization that is working to prevent the onset of climate change through promoting energy efficiency. Should you win, the amount will be deducted prior to you receiving the prize money and anonymously sent to Alliance to Save Energy.
If you win one of the prizes, how much of your winnings do you want sent to Alliance to Save Energy? Enter a dollar amount between $0 and $50.
Subjects entered number between 0 and 50.
Q13. Are you male or female?
a. Male
b. Female

Q14. What is your age in years?

Q15. What is the highest level of education you have completed?
a. Less than high school
b. High school or GED
c. Some college
d. 2-year college degree (Associates)
e. 4-year college degree (BA, BS)
f. Master's degree (MA, MS)
g. Doctoral degree (PhD)
h. Professional degree (MD, JD, DDS, etc)

Q16. Are you of Hispanic origin or descent, such as Mexican, Puerto Rican, Cuban, or other Spanish background?

a. Yes
b. No

Q17. Which of the following best describes your race?

a. White
b. African-American or Black
c. Asian
d. Asian-Pacific Islander
e. Native American
f. Other

Q18. Which of the following best describes your annual household income before taxes?

a. Less than $10,000
b. $10,000 - $19,999
c. $20,000 - $29,999
d. $30,000 - $39,999
e. $40,000 - $49,999
f. $50,000 - $74,999
g. $75,000 - $99,999
h. $100,000 - $149,999
i. $150,000 - $249,999
j. $250,000-$499,999
k. $500,000 and over

Q19. Which of the following best describes your current employment situation?

a. Employed full-time
b. Employed part-time
c. Retired
d. A homemaker
e. A student
f. Unemployed but looking for work
g. Unemployed and not looking for work
h. Disabled
i. Other

Q20. Which of the following best describes your political ideology?

a. Very Conservative
b. Conservative
c. Moderate
d. Liberal

e. Very Liberal
f. Other

Q21. Which of the following do you use to get news (select as many as apply)? [answer order was randomized]

a. CNN
b. Fox News
c. ABC News
d. CBS News
e. NBC News
f. PBS News
g. MSNBC
h. Wall Street Journal
i. New York Times
j. USA Today
k. Washington Post
l. Washington Times
m. US News
n. Other (please specify)

Thank you for completing the survey! We will notify you if you are one of the winners.

6-months follow-up survey – identical for all respondents

Q1. How much do you agree with each of the following statements? Strongly agree/agree/disagree/strongly disagree. [question order was randomized]

a. I am very interested in what’s going on with climate change policy.
b. As a society, we have a moral obligation to leave future generations at least as well off as we were.
c. It’s too late to prevent the harmful effects of global warming.
d. Climate scientists subject their findings to a sufficient amount of scrutiny.
e. Climate scientists gain financially from research that supports, rather than contradicts, the theory of global warming.
f. Climate scientists have ideological beliefs that prevent them from carrying out objective research.
g. Climate models are not nearly as reliable as climate scientists claim.

Q2. Which of the following gases is thought to contribute to global warming (select as many as apply)? [answer order was randomized]

a. Methane
b. Carbon dioxide
c. Argon
d. Nitrogen
e. Oxygen
f. Nitrous oxide
g. Ozone

Q3. Which of the following best demonstrates the albedo effect? [answer order was randomized]

a. Ice and other light surfaces reflecting sunlight back into the atmosphere.
b. Prevailing winds causing storm systems to rotate clockwise in the northern hemisphere.
c. Abnormally warm ocean temperatures causing more precipitation in some parts of the world.
d. Atmospheric gases trapping reflected sunlight and re-radiating some of it back toward the planet’s surface.

Q4. Which of the following is closest to the current carbon dioxide concentration in the atmosphere? [answer order was randomized]

a. 390-395 parts per million
b. 270-275 parts per million
c. 545-550 parts per million
d. 195-200 parts per million

Q5. What percentage of <u>all scientists who are knowledgeable about global warming and climate change</u> do you think would agree with each of the following statements? (Number of scientists out of 100)

i. "Scientists can say with great certainty that global warming is a process that is already underway."
ii. "Scientists can say with great certainty that human activities are accelerating global warming."
iii. "There is enough scientific uncertainty about the rate and extent of global warming and climate change that there is no need for immediate policy decisions."

Q6. In your opinion, what is the probability that each of the following is true, out of 100%?

a. Global warming is a process that is already underway.
b. Human activities are accelerating global warming.
c. There is enough scientific uncertainty about the rate and extent of global warming and climate change that there is no need for immediate policy decisions.

Subjects entered number between 0 and 100.

Q7. By how many degrees Fahrenheit do you expect temperatures on earth to rise or fall by the year 2050, on average (a change of 1 degree Fahrenheit is about equal to a change of 0.56 degrees Celsius)? A negative number corresponds to a fall in temperatures, while a positive number corresponds to a rise in temperatures.

Subjects entered number between 0 and 100.

Q8. What do you think is the probability that the temperature will <u>*increase by at least*</u> 2.5 degrees Fahrenheit by 2050, <u>out of 100%</u>?

Subjects entered number between 0 and 100.

Q9. What do you think is the probability that the temperature will <u>*increase by at least*</u> 5 degrees Fahrenheit by 2050, <u>out of 100%</u>?

Subjects entered number between 0 and 100.

Q10. What do you think is the probability that the temperature will <u>*decrease by at least*</u> 2.5 degrees Fahrenheit by 2050, <u>out of 100%</u>?

Subjects entered number between 0 and 100.

Q11. What do you think is the probability that the temperature will <u>*decrease by at least*</u> 5 degrees Fahrenheit by 2050, <u>out of 100%</u>?
Subjects entered number between 0 and 100.

Q12. Excluding weddings and funerals, approximately how often did you go to religious services over the past 12 months?

a. Once a week or more
b. Almost every week
c. Once or twice a month
d. A few times a year
e. Never

Q13. Outside of religious services, how often do you pray?

a. Several times a day
b. Once a day
c. A few times a week
d. Once a week or less
e. Never

Q14. Which of the following best describes your religious preference?

a. Protestant
b. Catholic
c. Jewish
d. Muslim
e. Mormon
f. Some other religion
g. No religion

Q15. During the past 5 years, did you usually vote in national, state, and local elections?

a. Yes
b. No

Q16. Are you currently registered to vote?

a. Yes
b. No
c. Don't know

Q17. Do you consider yourself a Republican, a Democrat, an independent, or something else?

a. Republican
b. Democrat
c. Independent
d. Libertarian
e. Something else

Q18. Do you favor or oppose an amendment to the U.S. Constitution banning marriage between two people who are the same sex?

a. Favor
b. Oppose

c. Neither favor nor oppose

Q19. Do you favor or oppose raising federal income taxes for people who make $200,000 per year or *more*?

a. Favor
b. Oppose
c. Neither favor nor oppose

Q20. Do you favor or oppose the U.S. government paying for all of the cost of prescription drugs for senior citizens who are living on very little income?

a. Favor
b. Oppose
c. Neither favor nor oppose

Q21. Do you favor or oppose the U.S. government paying for all necessary medical care for all Americans?

a. Favor
b. Oppose
c. Neither favor nor oppose

Q22. Imagine that the U.S. government suspects a person in the United States of being a terrorist. Do you favor or oppose the government being able to put this person in prison for months without ever bringing the person to court and charging him or her with a crime?

a. Favor
b. Oppose
c. Neither favor nor oppose

Q23. Do you favor or oppose the U.S. government making it possible for illegal immigrants to become U.S. citizens?

a. Favor
b. Oppose
c. Neither favor nor oppose

Q24. Would you vote in favor of a law that significantly reduced greenhouse gas emissions in the US and raised your household's annual electricity bill by $150?

a. Yes
b. No
c. Don't know

Q25. Would you vote in favor of a law that significantly reduced greenhouse gas emissions in the US and raised your household's annual electricity bill by $300?

a. Yes
b. No
c. Don't know

Q26. About how much did you donate to charity, including religious organizations, in the past year?
____ dollars

Q27. Which of the following do you use to get news (select as many as apply)? [answer order was randomized]

a. CNN
b. Fox News
c. ABC News
d. CBS News
e. NBC News
f. PBS News
g. MSNBC
h. Wall Street Journal
i. New York Times
j. USA Today
k. Washington Post
l. Washington Times
m. US News
n. Other (please specify)

Q28. After completing the survey, you will be entered in a drawing for one of six $50 prizes. You have the option to send part of your winnings to Alliance to Save Energy, a nonprofit organization that is working to prevent the onset of climate change through promoting energy efficiency. Should you win, the amount will be deducted prior to you receiving the prize money and anonymously sent to Alliance to Save Energy.
If you win one of the prizes, how much of your winnings do you want sent to Alliance to Save Energy? Enter a dollar amount between $0 and $50.

Subjects entered number between 0 and 50.

Thank you for completing the survey! We will notify you if you are one of the winners.
