# Supplementary material for: The Effect of Information Provision on Public Consensus about Climate Change
Source: PLoS One. 2016 Apr 11;11(4):e0151469. doi: 10.1371/journal.pone.0151469 (PMC4827814; doi:10.1371/journal.pone.0151469)
Supplement: S4 Text — (PDF) [file pone.0151469.s004.pdf]

S4. Summary Statistics

Table A presents the summary statistics for our study sample and p-values of t tests for differences in means between the treated and control groups. Our baseline treatment groups were well-balanced: respondents in the control or treatment groups did not significantly differ across any of the observable demographic characteristics. We do observe some selection into the follow-up survey: respondents who were older, employed, in the lower and middle income categories, and those with higher education were more likely to respond to the follow-up survey. Respondents in the "other" race category were marginally more likely to follow up.

Table A: Summary Statistics

Variable:	Average of Group [# Obs in Brackets]				p-value (t test of difference in means)		
	Control	HI Treatment	SI Treatment	Followed Up	Control v. All Treated	HI v. SI Treatment	Followed Up v. Did Not
Age	46.5	47.6	46.9	49.2	0.456	0.567	0.000***
	[431]	[423]	[425]	[797]			
% Male Respondents	52.0%	52.9%	56.9%	55.2%	0.315	0.236	0.239
	[433]	[431]	[434]	[808]			
% Asian Respondents	5.8%	3.2%	4.4%	5.0%	0.108	0.386	0.281
	[433]	[431]	[434]	[808]			
% Black Respondents	5.5%	9.3%	6.7%	6.3%	0.109	0.159	0.126
	[433]	[431]	[434]	[808]			
% White Respondents	84.8%	84.2%	86.4%	86.1%	0.789	0.365	0.190
	[433]	[431]	[434]	[808]			
% Other Racial Category	5.5%	5.1%	3.2%	3.8%	0.264	0.167	0.084*
	[433]	[431]	[434]	[808]			
% Employed	59.6%	57.5%	58.4%	60.8%	0.567	0.793	0.035**
	[399]	[402]	[406]	[760]			
% Low Income (<$40,000)	36.6%	36.0%	32.6%	31.1%	0.419	0.282	0.000***
	[432]	[430]	[433]	[807]			
% Mid Income ($40,000 - $99,999)	49.1%	47.7%	51.0%	52.4%	0.922	0.323	0.004***
	[432]	[430]	[433]	[807]			
% High Income (>$100,000	14.4%	16.3%	16.4%	16.5%	0.354	0.963	0.306
	[432]	[430]	[433]	[807]			
% Conservative	31.7%	33.0%	28.5%	32.2%	0.720	0.149	0.251
	[432]	[427]	[432]	[804]			
% Liberal	25.0%	24.1%	22.0%	23.3%	0.437	0.459	0.630
	[432]	[427]	[432]	[804]			
% High Education (4-year college or higher)	44.3%	46.0%	42.0%	47.0%	0.925	0.244	0.009***
	[422]	[417]	[421]	[790]			
Trust Scientists Index	2.053	2.028	1.956	1.979	0.405	0.404	0.217
	[433]	[431]	[434]	[808]			
% High Knowledge (3+ correct)	0.524	0.541	0.509	0.525	0.984	0.356	0.993
	[433]	[431]	[434]	[808]			

Notes: Mean values for groups of respondents. Number of observations in a given group for a given demographic category in brackets. p-values and significance levels based on t-tests of differences: * 10 percent, ** 5 percent, *** 1 percent.
