# Supplementary material for: The Effect of Information Provision on Public Consensus about Climate Change
Source: PLoS One. 2016 Apr 11;11(4):e0151469. doi: 10.1371/journal.pone.0151469 (PMC4827814; doi:10.1371/journal.pone.0151469)
Supplement: S5 Text — (PDF) [file pone.0151469.s005.pdf]

S5. Analysis of Skepticism

Almost two-thirds (65%) of our treated group did not think the information from the scientist survey was accurately representing the views of all scientists knowledgeable about climate change. The skepticism largely stems from the concern that the scientists polled ("US scientists who published in top journals") were not representative of *all* scientists knowledgeable about climate change: 85% of skeptical respondents chose that as a reason for thinking that the information was inaccurate. Only about 20% of the skeptical respondents thought that participating scientists misstated their true views.

Figure A plots the distributions of the individual responses as to what percentage of *all* scientists knowledgeable about climate change would agree with the statement that "Scientists can say with great certainty that human activities are accelerating global warming." We compare the responses to this question by members of the control group to members of the HI treatment group who thought the information presented to them in the survey was inaccurate. We find that, relative to the control group, the treated skeptics expect there to be significantly less consensus on climate change being caused by humans among *all* (representative) scientists.

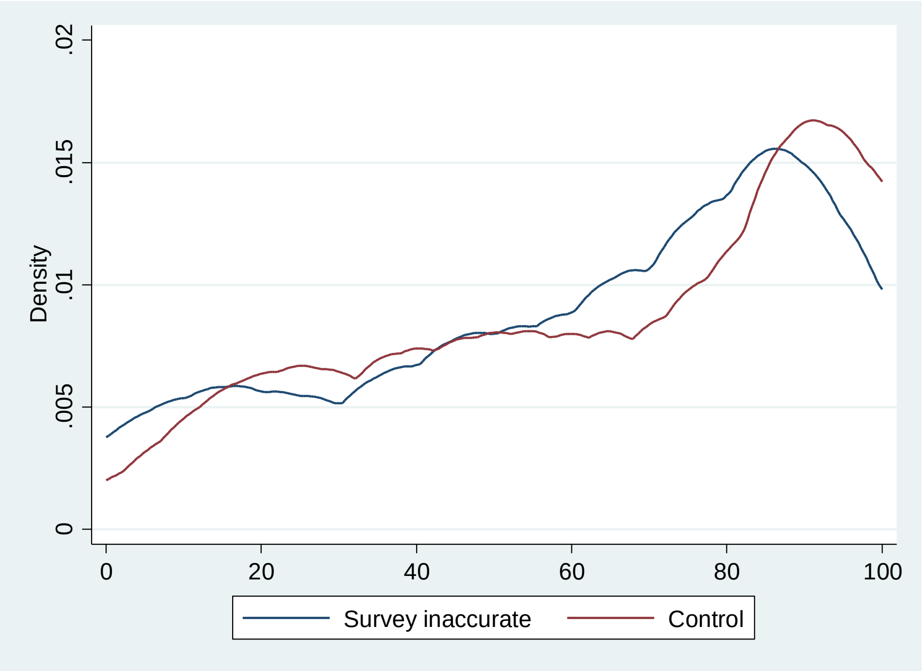

Figure A: Distribution of beliefs about what percent of *all* scientists knowledgeable about climate change would agree with the statement that "Scientists can say with great certainty that human activities are accelerating global warming."

Table A reports the estimates from a probit regression of the effects of individual characteristics on whether the information from the survey of scientists was perceived as accurate. We find that the unemployed are significantly more likely to be skeptical of the information from the survey. On the other hand, liberals and moderates, and those who trust scientists (Column 2) are more

likely to perceive the survey as accurate. Previous knowledge about climate change (Column 3) does not significantly impact skepticism in our sample.

Table A: Determinants of skepticism

Outcome variable:	Survey Accurate Dummy		
	(1)	(2)	(3)
Age	0.001	0.014	0.001
	(0.018)	(0.018)	(0.018)
Age Squared	0.00003	-0.0001	0.00003
	(0.0002)	(0.0002)	(0.0002)
Male	-0.084	-0.118	-0.078
	(0.155)	(0.151)	(0.150)
Asian	-0.373	-0.484	-0.392
	(0.674)	(0.669)	(0.676)
Black	-0.608	-0.74	-0.63
	(0.533)	(0.538)	(0.544)
White	-0.482	-0.611	-0.496
	(0.585)	(0.585)	(0.590)
Other	-0.108	-0.271	-0.127
	(0.579)	(0.557)	(0.589)
Unemployed	-0.993**	-0.953**	-1.001**
	(0.408)	(0.481)	(0.412)
Out of the Labor Force	-0.211	-0.145	-0.213
	(0.137)	(0.157)	(0.136)
Middle Income	0.051	-0.02	0.051
	(0.114)	(0.116)	(0.114)
High Income	0.188	0.23	0.184
	(0.169)	(0.177)	(0.171)
Moderate	0.451**	0.375**	0.445**
	(0.187)	(0.185)	(0.188)
Liberal	0.869***	0.614***	0.859***
	(0.161)	(0.171)	(0.160)
High education	0.07	0.004	0.065
	(0.101)	(0.114)	(0.099)
Trust Scientists		0.287***	
		(0.060)	
Knowledge			0.021
			(0.050)
Observations	399	399	399
R-squared	0.074	0.123	0.074

Notes: Robust standard errors clustered at the state level in parentheses. Middle income is $40,000-$99,999. High income is $100,000 and above. Out of the labor force includes: homemakers, students, disabled, and retired. High education refers to 4-year college degree or above. Significance levels: * 10 percent, ** 5 percent, *** 1 percent.
